# Vulcanite Work

**Published:** 1862-04

**Authors:** Geo. B. Snow


VULCANITE WORK.—*By Geo. B. Snow.*—There may be some of the readers of the Dental Cosmos, who use the vulcanite base, who would be glad to hear of a means of ascertaining the exact quantity of rubber which would be necessary for a certain case. I have used the following process during the past three months, and would recommend it as an excellent substitute for "guess work." I use sheet wax instead of gutta-percha, and am thus enabled to form my model plate entirely of one substance. On taking this wax plate from the mould, preparatory to packing the rubber, I strip off the thick foil which covers its palatal side, and weigh the wax; being careful to save all of it that was in the mould. I then place the wax on the same side of the scales with the weights, and balance them with rubber. This is of course twice the weight of the wax, which I have found to be an equal amount in bulk. It is as well to add three or four grains more of rubber; but it will nearly always be found in the gateways. If both parts of the flask are thoroughly warmed before they are packed, they can generally be brought together without the further application of heat.

Those who use the sheet gutta-percha can arrive at the same result by partially filling a very narrow glass vessel with water, and noting how much the level of the water is raised by submerging the plate. An amount of rubber sufficient to raise the water to the same level will suffice to fill the mould. Care must be taken, in this process, not to lose any of the water.—*Dental Cosmos.*

